# A Versatile Hybrid Mock Circulation for Hydraulic Investigations of Active and Passive Cardiovascular Implants

**DOI:** 10.1097/MAT.0000000000000851

**Published:** 2018-07-23

**Authors:** Anastasios Petrou, Marcus Granegger, Mirko Meboldt, Marianne Schmid Daners

**Affiliations:** From the * Product Development Group Zurich, Department of Mechanical and Process Engineering, ETH Zurich, Zurich, Switzerland; † Pediatric Heart Center, University Children’s Hospital, University of Zurich, Zurich, Switzerland.

**Keywords:** ventricular assist device, blood pump, numerical model of cardiovascular system, Fontan, physiologic control, biventricular support, total artificial heart, *in-vitro* testing

## Abstract

During the development process of active or passive cardiovascular implants, such as ventricular assist devices or vascular grafts, extensive *in-vitro* testing is required. The aim of the study was to develop a versatile hybrid mock circulation (HMC) which can support the development of such implants that have a complex interaction with the circulation. The HMC operates based on the hardware-in-the-loop concept with a hydraulic interface of four pressure-controlled reservoirs allowing the interaction of the implant with a numerical model of the cardiovascular system. Three different conditions were investigated to highlight the versatility and the efficacy of the HMC during the development of such implants: 1) biventricular assist device (BiVAD) support with progressive aortic valve insufficiency, 2) total artificial heart (TAH) support with increasing pulmonary vascular resistance, and 3) flow distribution in a total cavopulmonary connection (TCPC) in a Fontan circulation during exercise. Realistic pathophysiologic waveforms were generated with the HMC and all hemodynamic conditions were simulated just by adapting the software. The results of the experiments indicated the potential of physiologic control during BiVAD or TAH support to prevent suction or congestion events, which may occur during constant-speed operation. The TCPC geometry influenced the flow distribution between the right and the left pulmonary artery, which was 10% higher in the latter and led to higher pressures. Together with rapid prototyping methods, the HMC may enhance the design of implants to achieve better hemodynamics. Validation of the models with clinical recordings is suggested for increasing the reliability of the HMC.

## Abstract 2

Supplemental Digital Content is available in the text.

The typical development process of active and passive cardiovascular implants, such as ventricular assist devices and vascular grafts, consists of several steps. It starts with the *in-silico* modeling of the hydraulic properties to virtually test the implant, continues with the *in-vitro* testing to verify the *in-silico* results with physical models before testing the device *in vivo*. *In-vivo* testing allows the validation of the performance of the implant in animals before proceeding with clinical testing and application. While in terms of pressures and flows *in-silico* hydraulic models and boundary conditions can be adjusted as desired to mimic a realistic hemodynamic scenario, such an adjustment is more demanding with *in-vitro* setups, which are required to accurately imitate real conditions.

Conventional hydraulic mock circuits to investigate the hydraulic properties of cardiovascular implants consist of tubes, open and air-trapped reservoirs, valves, and cardiac simulators to simulate hemodynamic conditions.^1^ However, undesired effects because of the fluid inertance may occur when tubes and valves are used, especially in the case of *in-vitro* imitation of the cardiovascular system, which constitutes a very dynamic and complex environment. Thus, realistic waveforms of pressure and flow characteristics cannot be achieved at high fidelity. Furthermore, the versatility of such mock circuits is limited because hardware changes are required whenever different conditions are to be tested. Systems are thus required to evaluate different cardiovascular implants efficiently and in a versatile way with realistic hemodynamic waveforms.

Such systems have been presented earlier for the evaluation of left ventricular assist devices (LVADs). Besides others,^2–4^ Ochsner *et al.*^5^ used air and vacuum pressure-regulated reservoirs to mimic the left ventricular pressure (LVP) and the aortic pressure (AoP), which are computed by a numerical model of the cardiovascular system. This hybrid mock circulation (HMC) operates based on the hardware-in-the-loop (HIL) approach. It can be used to evaluate LVADs and their control algorithms. In HMCs, all components of the cardiovascular system are simulated numerically. The reservoirs, which are the main hardware parts of the system, form the interface between the numerical model of the cardiovascular system and the LVAD. The numerical model can be of almost unlimited complexity, for instance, to mimic any desired input impedance of the arterial vasculature,^6^ which cannot be achieved with a conventional hardware-based system. Furthermore, if any physiologic feedback control mechanisms of compliances, resistances, *etc.* are desired, the HIL interface provides advantages in terms of complexity; while with the numerical model the compliance value can be easily adjusted, in hardware-based loops, the amount of air in an air-trapped reservoir needs to be adjusted using a pneumatically controlled system for each reservoir.

In this study, the technology of the HMC developed earlier with two pressure reservoirs^5^ was extended to four reservoirs and the numerical model adapted according to the specific investigation. This versatile HMC allows to test complex active and passive cardiovascular implants, such as biventricular assist devices (BiVADs), total artificial hearts (TAHs), and total cavopulmonary connections (TCPCs) for Fontan patients, *i.e.*, patients with a single-functional ventricle who underwent the Fontan procedure, *i.e.*, the surgery where the caval veins are directly connected to the pulmonary arteries to palliate their symptoms yielding a TCPC.

Currently, 10–30% of LVAD recipients develop right ventricular (RV) failure,^7^ and in many cases, a BiVAD support treatment is followed. The outcomes of BiVAD support with rotary blood pumps have been worse than with LVAD support.^8,9^ The fluid balance between the pulmonary and the systemic circulation is challenging with two pumps running at constant speeds. Therefore, appropriate test setups to investigate new control methods of physical devices in an early development phase are required to verify *in-silico* methods to control and adapt the two pumps to each other and to the physiologic requirements of the circulation.

When the biventricular failure is treated with a TAH,^10^ the hydraulic performance of the device and its interaction with the cardiovascular system are crucial and should be evaluated at an early development phase using appropriate *in-vitro* facilities. The lack of neurohumoral cardiac response during TAH support constitutes a challenge for any physiologic adaptation of the device to the demands of the patient^11^ and the control of the left/right fluid balance remains a challenging topic.^12^ Finally, in the case of Fontan patients with a TCPC, no power is added to the blood on the subpulmonary side and any pressure drop due to the geometry of the TCPC impedes a sufficient venous return.^13^ Simulated flow fields and pressure losses in the TCPC need to be validated *in vitro* with realistic flows and vascular impedances.

The versatile HMC developed can be employed for evaluating the performance of BiVADs and TAHs and their control algorithms, when interacting with the cardiovascular system. Furthermore, it allows the assessment of the influence of the TCPC geometry on Fontan hemodynamics, such as the resulting pressure losses, under various pathophysiological conditions.

## Materials and Methods

In all three test cases presented, the same hardware setup was used and only the software part, *i.e.*, the numerical model of the cardiovascular system, was adapted. Both hardware and software parts are described below and in the Supplemental Digital Content in detail (http://links.lww.com/ASAIO/A308).

### Hardware

**Figure 1.**
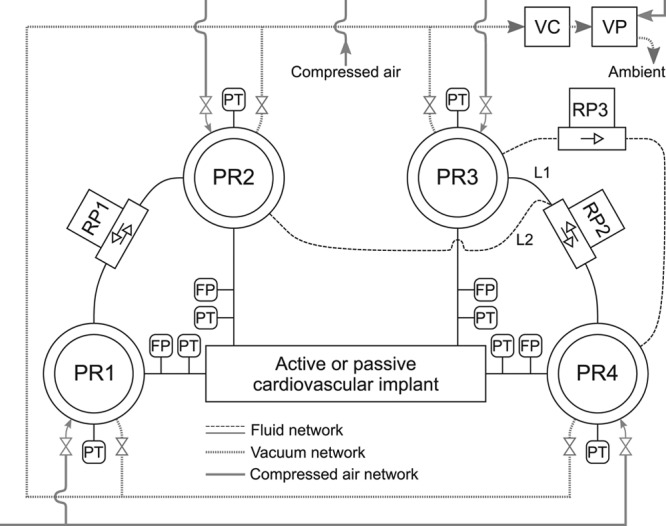
Scheme of the hardware parts of the versatile hybrid mock circulation developed. It consists of four pressure reservoirs (PR1–PR4), three reflux pumps (RP1–RP3), one VC, one VP, eight PTs, and four FPs. For the Fontan experiment, the dashed line (L2) replaces the solid line (L1). The RP3 is only used during the Fontan experiment. FPs, flow probes; PTs, pressure transducers; VC, vacuum chamber; VP, vacuum pump.

**Figure 1** depicts a schematic overview of the hardware parts of the HMC developed, divided into the hydraulic and the pneumatic system. The hydraulic system consists of four pressure reservoirs (PR1–PR4) whose pressures are controlled by using pressurized air and vacuum based on pressure measurements (PN2009, IFM Electronic GmbH, Essen, Germany). Additional hardware includes four ultrasound flow probes (CO.55/190, Sonotec Ultraschallsensorik Halle GmbH, Halle, Germany), four pressure transducers for monitoring inline pressures (TruWave, Edwards, Lifesciences, Irvine, CA, USA), and three reflux pumps (two Jabsco 18660 Series, Xylem Inc., NY, USA, and one S-pump, Xenios AG, Heilbronn, Germany). The pneumatic system consists of one vacuum pump (ZL112-K15LOUT-E26L-Q, SMC Pneumatics, Tokyo, Japan), one vacuum chamber and proportional solenoid valves, one inlet valve (PVQ33-5G-23-01F, SMC Pneumatics) for connecting each reservoir with the compressed air from the network supply, and two outlet valves (PVQ33-5G-40-01F, SMC Pneumatics) for connecting each reservoir with the vacuum chamber. These valves were controlled to achieve the desired pressures in the respective four reservoirs.

### Software

**Figure 2.**
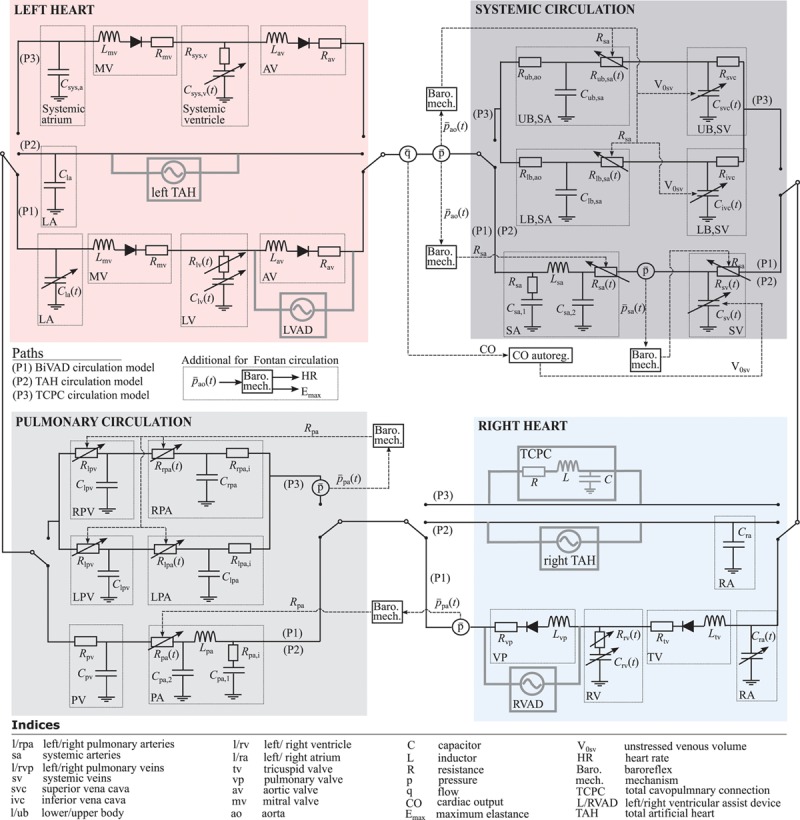
Electric analog of the numerical models of the cardiovascular system used in this study. Path 1 (P1) corresponds to the BiVAD test case, path 2 (P2) to the TAH test case, and path 3 (P3) to the TCPC test case. Gray lines indicate the interfaces of the respective implant to the numerical model. The additional control mechanisms for heart rate and maximum elastance are only used for the Fontan circulation based on Granegger *et al.*^15^ BiVAD, biventricular assist device; TAH, total artificial heart; TCPC, total cavopulmonary connection.

**Figure 2** presents the three different numerical models of the cardiovascular systems used in each test of this study. Each model consists of four main parts, the left heart (red), the right heart (blue), the pulmonary circulation (light gray), and the systemic circulation (dark gray). The arterial and venous systems were simulated by five- and three-element Windkessel models, respectively, resulting in different arterial input impedances. In the TAH configuration, no active ventricular models were employed. The lumped parameter models for the BiVAD and TAH cases were adopted from Colacino *et al.*^14^ Based on that model, control mechanisms for the unstressed venous volume, the systemic venous and arterial resistances, as well as the pulmonary arterial resistance, were implemented. For the univentricular cardiovascular system, the model was derived from Granegger *et al.*,^15^ which included control mechanisms for the systemic and pulmonary arterial resistance as well as the unstressed venous volume. Furthermore, heart rate (HR) and maximum elastance control mechanisms were incorporated, analogously to those reported.^14^ A detailed description of the model is provided in the Supplemental Digital Content (http://links.lww.com/ASAIO/A308).

### Active Cardiovascular Implant

Two HeartWare HVADs (Medtronic Inc., Minneapolis, MN, USA) were used for both the BiVAD and the TAH configuration experiments. To control the HVAD speed, an in-house speed controller was developed based on a control board (LAUNCHXL-F28069M) with two DC drive stage modules (BOOSTXL-DRV8305EVM) both from Texas Instruments (Dallas, TX, USA) for the two motors of the pump. In both BiVAD and TAH configuration experiments, the HVAD speed was either set at a constant value or controlled dynamically with a physiologic controller.

### Test Case 1: Biventricular Assist Device Support During Aortic Valve Insufficiency

**Figure 3.**
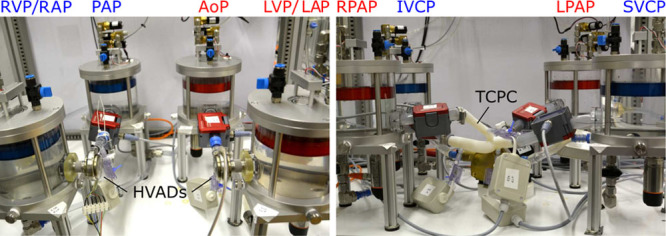
Left: Picture of the HMC during the BiVAD and TAH configuration experiments with the two HeartWare HVADs installed. Right: Picture of the HMC during the Fontan experiments with the three-dimensional-printed TCPC. AoP, aortic pressure; BiVAD, biventricular assist device; HMC, hybrid mock circulation; IVCP, inferior vena cava pressure; LAP, left atrial pressure; LPAP, left pulmonary arterial pressure; LVP, left ventricular pressure; PAP, pulmonary arterial pressure; RAP, right atrial pressure; RPAP, right pulmonary arterial pressure; RVP, right ventricular pressure; SVCP, superior vena cava pressure; TAH, total artificial heart; TCPC, total cavopulmonary connection.

A pathologic circulation of an adult patient with biventricular failure was simulated with reduced RV and LV ejection fractions of 30% and 20%, respectively.^9^ Both ventricles were supported with an HVAD whose speed was adjusted to yield a cardiac output (CO) of 5 L/min. **Figure 3** (left) depicts the HVAD configuration on the HMC. A resistance element was added at the outflow of the RVAD to reduce the flow by increasing the pressure head across the pump while keeping the operating speed between 1,800 and 4,000 rpm.^7^

In this study, we simulated the implantation of the RVAD into the RV, unlike another possibility that is commonly reported, which features RVAD implantation in the right atrium.^7^ To investigate the fluid balance between the systemic and the pulmonary vasculature during BiVAD support, we simulated a transition from mild to severe aortic insufficiency (AI), which seems to occur in a large number of patients supported by continuous-flow VADs.^16^ For this purpose, the resistance of the aortic valve was adapted during baseline conditions to result in regurgitant fractions of less than 30% and greater than 50%, respectively.^17^

This transition experiment was conducted twice. First, both HVADs were operated at a constant speed such that 5 L/min were supplied to the pulmonary and the systemic circulation in the baseline condition, where left atrial pressure (LAP) and right atrial pressure (RAP) were 12.5 and 2.7 mm Hg, respectively. For the second case, both HVADs were controlled to keep either preloads in a physiologic range. This algorithm was implemented to control the end-diastolic pressure (EDP) by a simple proportional controller, which increased the HVAD speed with increasing preload, as presented earlier^18^ and described in Equation 1:


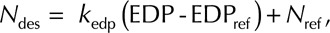


where *k*_edp_ is the proportional gain (rpm/mm Hg), EDP_ref_ is the set point EDP (mm Hg) defined during calibration, *i.e.*, while adjusting the reference speed *N*_ref_ (rpm).

### Test Case 2: Total Artificial Heart Configuration Support During Increase of the Pulmonary Vascular Resistance

The same model of the circulatory system of test case 1 was used for test case 2 but without ventricles. The two HVADs were now serving as a TAH configuration, thus pumping from a passive left or right atrium to the aorta or the pulmonary artery, respectively. A fivefold pulmonary vascular resistance (PVR) increase from 0.1 to 0.5 mm Hg·s/mL was applied^19^ to simulate the clinical condition of pulmonary hypertension, which may occur in VAD patients^20^ and lead to fluid imbalance problems. This experiment was conducted under two different control cases of the HVADs. First, they were operated at a constant speed, such that a pump flow and CO of 5 L/min resulted. Then, the experiment was repeated while the HVADs were controlled to keep the preloads in a physiologic range by controlling the LAP and RAP, *i.e.*, by applying the control structure of Equation 1 and by replacing the EDP with LAP and RAP, respectively, see **Figure 3** (left).

### Test Case 3: Total Cavopulmonary Connection Flow Distribution During Rest and Exercise

A rigid model of a TCPC was used as a passive cardiovascular implant. The geometry was derived from a patient who followed Fontan completion. For this purpose, cardiovascular magnetic resonance imaging datasets were acquired from the patient.^21^ The TCPC geometry was three-dimensional-printed with Polyamide 12. The three-dimensional-printed TCPC geometry was evaluated when coupled with a numerical model of a univentricular cardiovascular system.^15^ That model includes closed-loop baroreflex and metabolic reflexes to simulate exercise. Increased power loss in the TCPC during exercise conditions has been reported to greatly influence the clinical outcomes of Fontan patients^21^ and thus requires investigations to develop new solutions. In our study, a baseline condition at rest was compared with an exercise level of three metabolic equivalents of tasks (METs) by recording the pressures and flow distribution within the TCPC, see **Figure 3** (right).

## Results

### Test Case 1: Biventricular Assist Device Support During Aortic Valve Insufficiency

**Figure 4.**
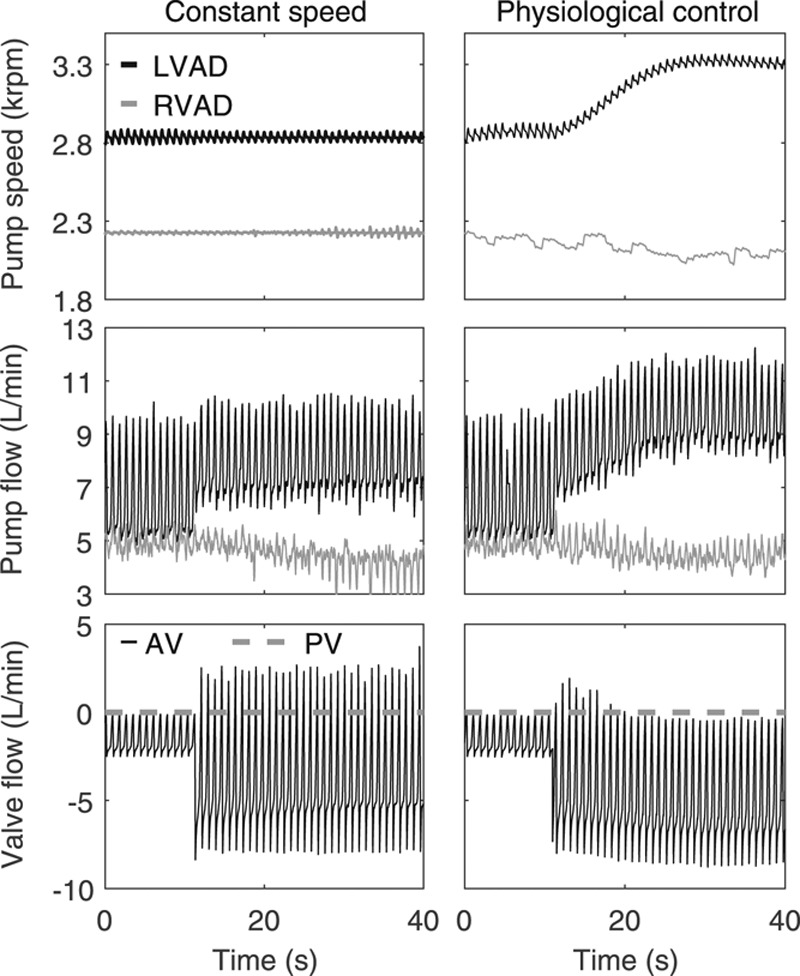
Measured and simulated signals during the biventricular support test case. The signals of the measured pump speed and flow as well as the simulated valve flows of the AV and PV are depicted. AV, aortic valve; LVAD, left ventricular assist device; PV, pulmonary valve; RVAD, right ventricular assist device.

**Figure 5.**
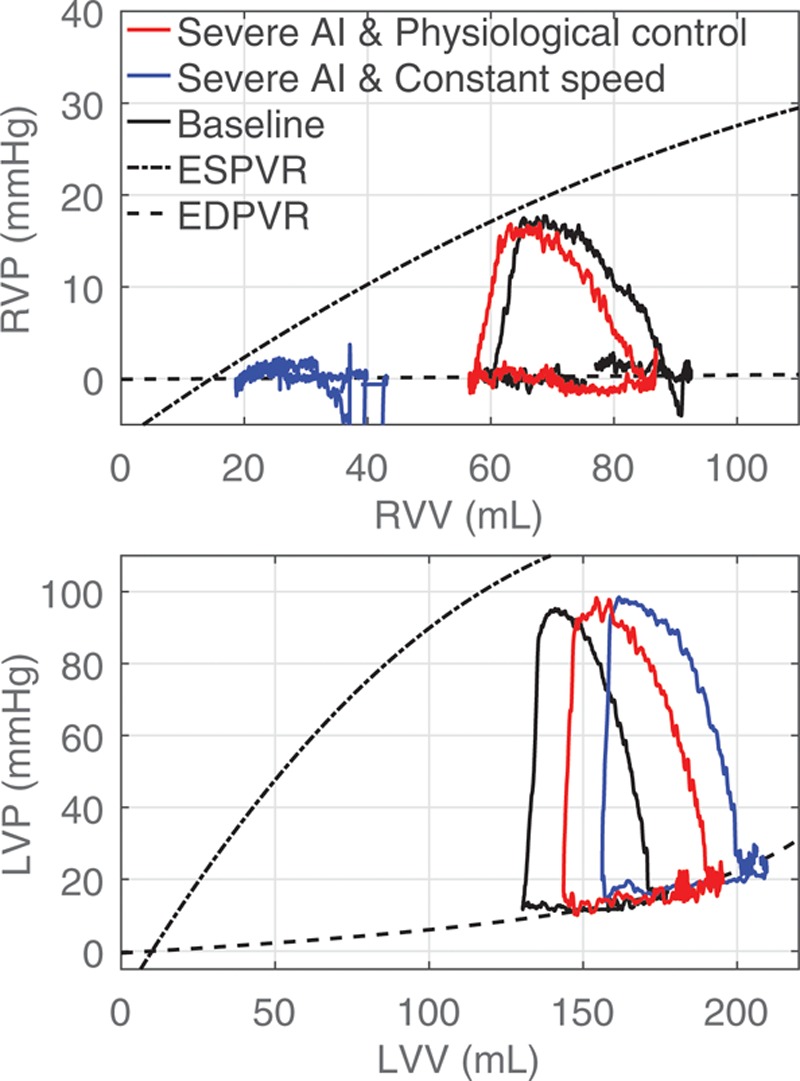
Pressure–volume loops of the right (RVP–RVV) and left ventricles (LVP–LVV) during the biventricular support test case. Loops during mild and severe AI are depicted. The end-diastolic (EDPVR) and end-systolic pressure–volume relationships (ESPVR) are also illustrated. AI, aortic insufficiency; LVP, left ventricular pressure; LVV, left ventricular volume; RVP, right ventricular pressure; RVV, right ventricular volume.

During constant-speed BiVAD support, the AI progression led to an increase in LV preload, with an LV EDP elevation from 15 to 24 mm Hg, respectively (**Figure 4**). That preload increase, in turn led to an LVAD PF increase from 6.2 to 7.5 L/min, but the corresponding CO decreased from 4.9 to 4 L/min due to the increased regurgitant flow. The RVAD PF decreased by 1 L/min due to the RV preload decrease, which resulted from the blood volume shift to the pulmonary circulation as well as the afterload increase. The RVAD speed remained at 2,200 rpm and as a result, excessive unloading with negative pressures and consequent RV suction occurred (see RV pressure–volume (PV) loops in blue in **Figure 5**). In the case of physiologically controlled pumps, the PS of the LVAD increased to 3,300 rpm and the RVAD PS decreased to 2,000 rpm during the AI progression. Negative pressures are also observed in **Figure 5** in the RV PV loops in black and red. The reason for those was the limited ability of the pneumatic pressure controllers of the hydraulic interface^5^ to accurately apply a positive pressure close to 0 mm Hg to the pressure tanks. Yet, they were not corresponding to suction. The LVEDP increased from 15 to 19 mm Hg at a CO of 4.9 and 4.5 L/min, respectively. The LVAD PF increased to 9.5 L/min, while the RVAD PF decreased to 4.5 L/min. The PV loops of the RV changed marginally, showing a slight preload decrease.

### Test Case 2: Total Artificial Heart Configuration Support During Increase of the Pulmonary Vascular Resistance

**Figure 6.**
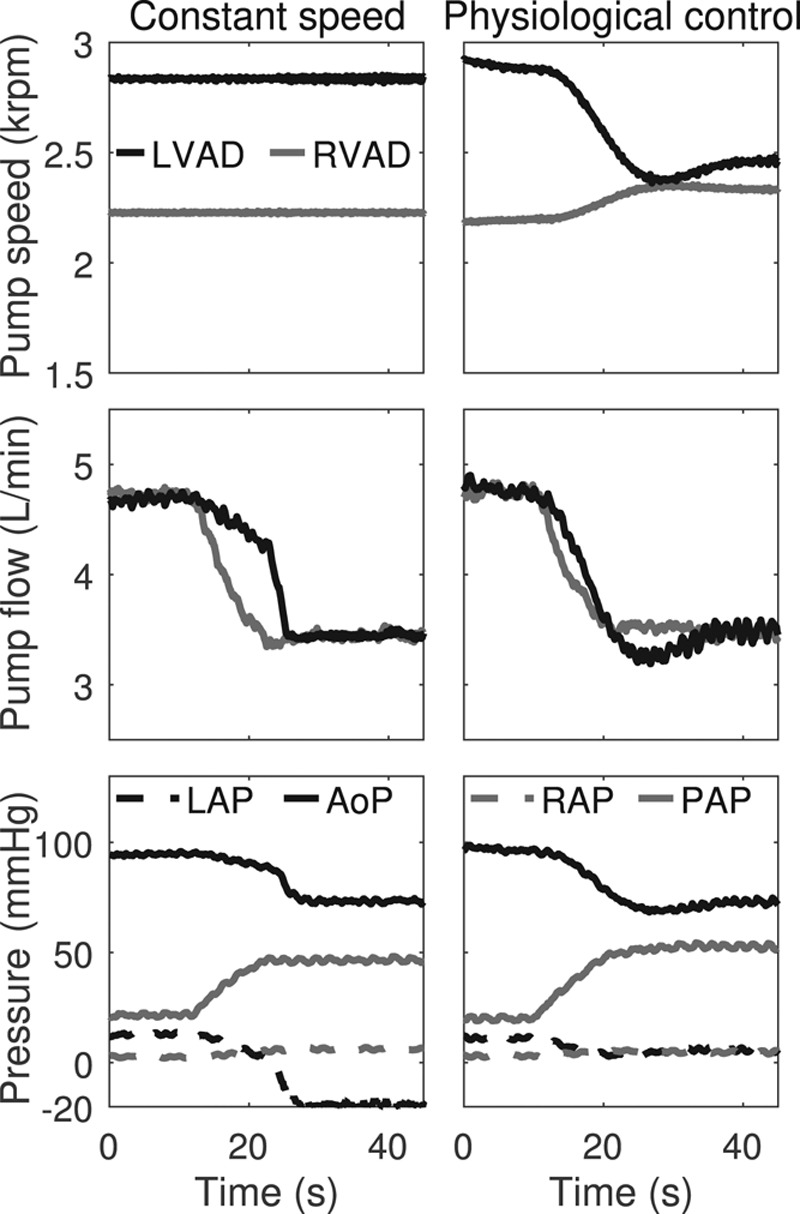
*In-vitro* performance of a TAH configuration consisting of two HeartWare HVADs and operating either at a constant speed or with physiologic control during an increase of PVR. The signals of the pump speeds and flows as well as of the LAP, the AoP, the RAP, and the PAP are depicted. AoP, aortic pressure; LAP, left atrial pressure; LVAD, left ventricular assist device; PAP, pulmonary arterial pressure; PVR, pulmonary vascular resistance; RAP, right atrial pressure; RVAD, right ventricular assist device; TAH, total artificial heart.

In the constant-speed case, the RVAD flow decreased due to the PVR increase and the lack of speed adaptation, which led to a preload decrease for the LVAD (**Figure 6**). The LVAD speed did not decrease and, therefore, negative LAPs occurred at t >25 sec. With physiologic control, the LVAD speed decreased by approximately 400 rpm and the RVAD speed increased by 200 rpm after the PVR increased, thus keeping an equal pump flow between the LVAD and the RVAD. The RAP and LAP remained almost constant, whereas the AoP decreased and the pulmonary arterial pressure (PAP) increased.

### Test Case 3: Total Cavopulmonary Connection Flow Distribution During Rest and Exercise

**Figure 7.**
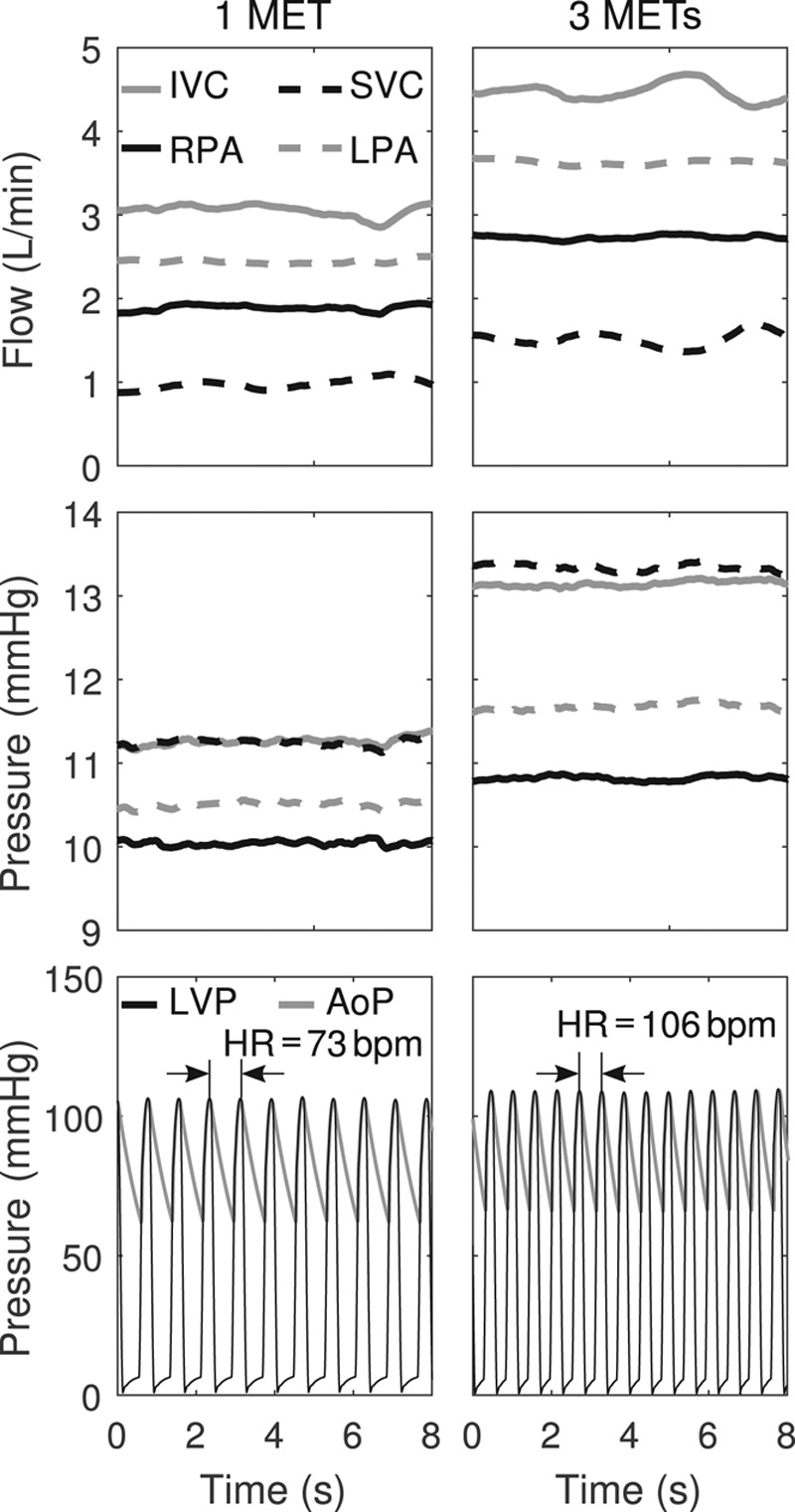
*In-vitro* results of the TCPC when interacting with the simulated Fontan circulation under 1 (left) and 3 (right) METs. The flows and pressures for the IVC and SVC, as well as the LPA and RPA, are depicted. The LVP and AoP are also shown in the bottom plots, as well as the HR increase from rest to exercise. AoP, aortic pressure; HR, heart rate; IVC, inferior vena cava; LPA, left pulmonary artery; LVP, left ventricular pressure; METs, metabolic equivalents of tasks; RPA, right pulmonary artery; SVC, superior vena cava; TCPC, total cavopulmonary connection.

At rest, the flow of the inferior vena cava (IVC) was three times greater than the one of the superior vena cava (SVC), see **Figure 7**. Due to the asymmetric geometry of the TCPC, the left pulmonary arterial (LPA) flow equaled 2.4 L/min and was 0.5 L/min higher than the right pulmonary arterial (RPA) flow. The IVC and SVC pressures were equal, while the LPA pressure was 0.5 mm Hg higher than the RPA pressure. These differences were also observed during the exercise condition: The IVC flow increased by 1.5 L/min but remained three times larger than the one of the SVC. The difference between LPA and RPA slightly increased up to 0.8 L/min, while the LPA flow equaled 3.6 L/min, thus keeping the flow ratio equal to that observed in rest conditions, *i.e.*, approximately 55% for LPA and 45% for RPA flow. The SVC and IVC pressures increased by approximately 2 mm Hg and they remained equal with each other. The LPA and RPA pressures increased by approximately 1 mm Hg, indicating a slightly elevated pressure drop during exercise. The LVP and AoP signals show that the HR increased from around 75 bpm at rest to around 105 bpm during exercise.

During all the experiments, the root-mean-square errors between the pressures computed by the numerical models and the pressures applied within the pressure reservoirs remained below 3 mm Hg.

## Discussion

In this study, a new HMC was presented that allows the evaluation of the performance of complex active and passive cardiovascular implants. It is able to accurately apply the pressures computed by numerical models to the hydraulic interface used, thus enabling a reliable interaction between the implant and the model. It offers a high flexibility during testing, as various clinical scenarios can be simulated simply by varying parameters of the model while avoiding hardware interventions. Thus, the performance of active implants and their control algorithms, as well as that of passive implants, can be evaluated before *in-vivo* testing. The flexibility and versatility of this HMC were proven with three test cases which required only software adjustments and the exchange of the device to be evaluated.

The principle of operation of the HMC developed is considered superior to existing approaches. The Donovan mock circulation, which is the best-known conventional system, was developed in 1975 and was recently used to evaluate the SynCardia TAH *in vitro*.^22^ It constitutes a pure hardware system that does not use any numerical model of the cardiovascular system. In contrast, in semihybrid systems, some of the components of the cardiovascular system are represented by physical components, such as tubes and tanks to mimic resistances, inertances, and compliances. Timms *et al.*^1^ introduced such a system to evaluate BiVAD cases. The ventricles are imitated by pneumatically-actuated chambers, equipped with solenoid valves that control the inflow and outflow of the pressurized air. A passive diastolic filling of the ventricles is simulated by venting these valves. As a result, the system relies on the inherent compliance of the trapped air, which may limit the generation of high-frequency, physiologic waveforms. Arterial and pulmonary Windkessel components were similarly imitated by proportional-controlled pinch valves to adjust resistances and air-trapped reservoirs with adaptable air volume to adjust the compliances. Tubes and connections are inevitable in such a system. The fluid inertia, which is an important contributor to vascular input impedances,^23^ thus cannot be adjusted as desired. Similar approaches have been presented by Schampaert *et al.*,^24^ who implemented positive displacement pumps to represent the ventricles and a polyurethane tube to mimic the elastic aortic properties or by Ruiz *et al.*,^25^ who used rubber bellows actuated by positive displacement pumps to model the atria and ventricles. Such semihybrid systems have also been used to evaluate a mechanical circulatory support (MCS) device for the Fontan circulation.^26^

In mock loops employing physical models of heart valves, an adjustment of the desired regurgitant fraction of an insufficient valve is cumbersome because it must be mechanically induced. In our setup, the resistance of the valves toward backflow was numerically adjusted (by a change of a parameter in software) in such a way that the amount of regurgitation matched the one recorded clinically.^17^ Furthermore, suction events with a realistic morphology in pump signals cannot be achieved in passive mock loops, which in our case was possible by using an approach developed earlier.^27^ However, investigation and testing of devices under such conditions are crucial because these are realistic worst-case scenarios, for instance, bearings of rotary blood pumps. Otherwise, such events can only be tested with less realistic environments or *in-vivo* trials.

Clinical-use cases during BiVAD and TAH support were investigated in the HMC presented; namely experiments with AI progression during BiVAD and PVR increase during TAH support. When operating these devices at constant speed, the problem of the fluid imbalance between systemic and pulmonary circulation was reproduced. Such conditions may lead to suction and pulmonary or systemic venous congestion. The conditions simulated matched well the published results of animal experiments under BiVAD support with and without physiologic control.^12^ We showed that the implementation of simple physiologic controllers can mitigate the risk of suction or congestion and create more physiologic conditions during BiVAD and TAH therapy. However, long-term, implantable pressure sensors are required for any clinical implementation of these advancements, whose development is still ongoing.^28,29^

The investigation of TCPC properties under realistic hemodynamic conditions, for instance at rest and exercise, is necessary to verify the results of *in-silico* studies with computational fluid dynamics.^21^ The combination of closed-loop baroreflex functionality and physical hydraulic properties of complex geometric TCPC structures provides a unique insight into their interaction. Employing rapid prototyping techniques, TCPC geometries of magnetic resonance (MR)/computed tomography (CT) images can be manufactured,^21^ and pressures as well as flow distributions within the TCPC can be investigated at a fast pace under various conditions. Therefore, the HMC offers a novel, reliable testing environment for passive cardiovascular implants and the assessment of their hydraulic properties in combination with their physiologic effects.

Apart from the cases presented, the HMC developed can be used for other experiments, such as evaluating artificial or mechanical valves, other grafts with multiple in- or outlets (*e.g.*, prosthetic replacements of the aortic arch) as well as for evaluating the use of MCS devices in Fontan patients or the BiVAD case with an RVAD pumping from the right atrium to the pulmonary artery. In general, this versatile HMC can be used for any case where one to four pressures interact with an implant.

## Limitations

A main limitation of this study is the lack of validation of the numerical models used with clinical observations. The model^14^ has been validated for investigating a physiologic heart under pre- and afterload changes. By modifying that model for our test cases, a new validation is required. However, due to the lack of clinical data, this constitutes a challenging topic. Only for the circulation with biventricular failure^9^ and for the simulation of AI^17^ data were available and matched the simulated conditions. Despite that, reasonable qualitative results were obtained, which matched previous animal studies.^12^ Future study should be focused on validating these numerical models.

## Conclusion

With its unique versatility and flexibility, the HMC presented constitutes a valuable tool for researchers that supports the development and investigation of complex active or passive cardiovascular implants such as TAHs and TCPCs. Its principle of operation allows for the generation of realistic pathophysiologic signal waveforms and the simulation of various clinical conditions. Thus, new devices and their control algorithms can be evaluated extensively at an early stage of development. The combination of rapid testing of TCPC geometries with such an HMC revealed unequal flow distributions and high pressure drops at the TCPCs. Such information is crucial and may support to optimize the TCPC design preoperatively.

## Acknowledgment

The authors thank the financial support by the Stavros Niarchos Foundation. This study is part of the Zurich Heart project under the umbrella of University Medicine Zurich. The authors also thank the technical support of Sara Mettler for the development of the control unit for the HVAD and of Luca Arpagaus for the development of the mock circulation. In addition, the authors thank Axel Krieger and Dominik Siallagan from Sheikh Zayed Institute for Surgical Innovation, Children’s National Medical Center, Washington, DC, for providing the 3D-printed TCPC geometry.

## Supplementary Material

## References

[1] Timms D Hayne M McNeil K Galbraith A: A complete mock circulation loop for the evaluation of left, right, and biventricular assist devices. Artif Organs 2005 29: 564–572. 15982285 10.1111/j.1525-1594.2005.29094.x

[2] Darowski M Kozarski M Ferrari G A new hybrid (hydro-numerical) model of the circulatory system. Bull PolAcad Sci - Tech Sci 2013 61: 993–1003.

[3] Misgeld BJE Rüschen D Schwandtner S Heinke S Walter M Leonhardt S: Robust decentralised control of a hydrodynamic human circulatory system simulator. Biomed Signal Process Control 2015 20: 35–44.

[4] Nestler F Bradley AP Wilson SJ Timms DL Frazier OH Cohn WE A hybrid mock circulation loop for a total artificial heart. Artif Organs 2014 38: 775–782. 25234760 10.1111/aor.12380

[5] Ochsner G Amacher R Amstutz A A novel interface for hybrid mock circulations to evaluate ventricular assist devices. IEEE Trans Biomed Eng 2013 60: 507–516. 23204266 10.1109/TBME.2012.2230000

[6] Westerhof N Lankhaar JW Westerhof BE The arterial Windkessel. Med Biol Eng Comput 2009 47: 131–141. 18543011 10.1007/s11517-008-0359-2

[7] Krabatsch T Montalto A Loforte A Musumeci F Krabatsch T Slaughter MS Biventricular circulatory support with two implantable continuous-flow pumps. in Mechanical Circulatory Support in End-Stage Heart Failure. 2017, pp. Switzerland, Cham: Springer International Publishing, 305–311.

[8] Kirklin JK Pagani FD Kormos RL Eighth annual INTERMACS report: Special focus on framing the impact of adverse events. J Heart Lung Transplant 2017 36: 1080–1086. 28942782 10.1016/j.healun.2017.07.005

[9] Krabatsch T Potapov E Stepanenko A Biventricular circulatory support with two miniaturized implantable assist devices. Circulation 2011 124(11 suppl): S179–S186. 21911810 10.1161/CIRCULATIONAHA.110.011502

[10] Sale SM Smedira NG Total artificial heart. Best Pract Res Clin Anaesthesiol 2012 26: 147–165. 22910087 10.1016/j.bpa.2012.04.002

[11] Abe Y Chinzei T Mabuchi K Physiological control of a total artificial heart: conductance- and arterial pressure-based control. J Appl Physiol 1998 84: 868–876. 9480945 10.1152/jappl.1998.84.3.868

[12] Gregory SD Stevens MC Pauls JP *In vivo* evaluation of active and passive physiological control systems for rotary left and right ventricular assist devices. Artif Organs 2016 40: 894–903. 26748566 10.1111/aor.12654

[13] de Zélicourt DA Kurtcuoglu V: Patient-specific surgical planning, where do we stand? The example of the Fontan procedure. Ann Biomed Eng 2016 44: 174–186. 26183962 10.1007/s10439-015-1381-9

[14] Colacino FM Moscato F Piedimonte F Arabia M Danieli GA Left ventricle load impedance control by apical VAD can help heart recovery and patient perfusion: A numerical study. ASAIO J 2007 53: 263–277. 17515714 10.1097/MAT.0b013e31805b7e39

[15] Granegger M Schweiger M Schmid Daners M Meboldt M Hübler M Cavopulmonary mechanical circulatory support in Fontan patients and the need for physiologic control: A computational study with a closed-loop exercise model. Int J Artif Organs 2018 41: 261–268. 29521133 10.1177/0391398818762359

[16] Deo SV Sharma V Cho YH Shah IK Park SJ De novo aortic insufficiency during long-term support on a left ventricular assist device: A systematic review and meta-analysis. ASAIO J 2014 60: 183–188. 24399060 10.1097/MAT.0000000000000042

[17] Zoghbi WA Enriquez-Sarano M Foster E; American Society of Echocardiography: Recommendations for evaluation of the severity of native valvular regurgitation with two-dimensional and Doppler echocardiography. J Am Soc Echocardiogr 2003 16: 777–802. 12835667 10.1016/S0894-7317(03)00335-3

[18] Petrou A Monn M Meboldt M Schmid Daners M A novel multi-objective physiological control system for rotary left ventricular assist devices. Ann Biomed Eng 2017 45: 2899–2910. 28900761 10.1007/s10439-017-1919-0

[19] Khalil HA Kerr DT Franchek MA Continuous flow total artificial heart: modeling and feedback control in a mock circulatory system. ASAIO J 2008 54: 249–255. 18496274 10.1097/MAT.0b013e3181739b70

[20] Zimpfer D Zrunek P Sandner S Post-transplant survival after lowering fixed pulmonary hypertension using left ventricular assist devices. Eur J Cardiothorac Surg 2007 31: 698–702. 17289396 10.1016/j.ejcts.2006.12.036

[21] Siallagan D Loke YH Olivieri L Virtual surgical planning, flow simulation, and 3-dimensional electrospinning of patient-specific grafts to optimize Fontan hemodynamics. J Thorac Cardiovasc Surg 2018 155: 1734–1742. 29361303 10.1016/j.jtcvs.2017.11.068 PMC5860962

[22] Crosby JR DeCook KJ Tran PL Physiological characterization of the SynCardia total artificial heart in a mock circulation system. ASAIO J 2015 61: 274–281. 25551416 10.1097/MAT.0000000000000192 PMC4414704

[23] Stergiopulos N Westerhof BE Westerhof N Total arterial inertance as the fourth element of the Windkessel model. Am J Physiol 1999 276: H81–H88. 9887020 10.1152/ajpheart.1999.276.1.H81

[24] Schampaert S Pennings KA van de Molengraft MJ Pijls NH van de Vosse FN Rutten MC A mock circulation model for cardiovascular device evaluation. Physiol Meas 2014 35: 687–702. 24622168 10.1088/0967-3334/35/4/687

[25] Ruiz P Rezaienia MA Rahideh A Keeble TR Rothman MT Korakianitis T *In vitro* cardiovascular system emulator (bioreactor) for the simulation of normal and diseased conditions with and without mechanical circulatory support. Artif Organs 2013 37: 549–560. 23758568 10.1111/aor.12109

[26] Rodefeld MD Coats B Fisher T Cavopulmonary assist for the univentricular Fontan circulation: Von Kármán viscous impeller pump. J Thorac Cardiovasc Surg 2010 140: 529–536. 20561640 10.1016/j.jtcvs.2010.04.037 PMC2924921

[27] Ochsner G Amacher R Schmid Daners M: Emulation of ventricular suction in a hybrid mock circulation. In: Proceedings of the 12th European Control Conference, 2013 pp. 2013 New York City, NY 3108–3112, 31083112.

[28] Staufert S Hierold C Novel sensor integration approach for blood pressure sensing in ventricular assist devices. Procedia Eng 2016 168: 71–75.

[29] Tchantchaleishvili V Luc JGY Cohan CM Clinical implications of physiologic flow adjustment in continuous-flow left ventricular assist devices. ASAIO J 2017 63: 241–250. 28459742 10.1097/MAT.0000000000000477

