# Supplementary material for: A Versatile Hybrid Mock Circulation for Hydraulic Investigations of Active and Passive Cardiovascular Implants
Source: ASAIO J. 2018 Jul 23;65(5):495–502. doi: 10.1097/MAT.0000000000000851 (PMC6615934; doi:10.1097/MAT.0000000000000851)
Supplement: Supplementary file 1 [file mat-65-495-s001.docx]

Supplementary material

A versatile hybrid mock circulation for hydraulic investigations of active and passive cardiovascular implants

Anastasios Petrou,† Marcus Granegger,‡ Mirko Meboldt,† Marianne Schmid Daners†

**Affiliations: From the †pd|z Product Development Group Zurich, Department of Mechanical and Process Engineering, ETH Zurich, Zurich, Switzerland; the** ‡**Pediatric Heart Center, University Children’s Hospital, University of Zurich, Zurich, Switzerland;**

**Running Title: A versatile hybrid mock circulation**

Corresponding Author: Dr. Marianne Schmid Daners, [marischm@ethz.ch](mailto:marischm@ethz.ch), ETH Zurich, CLA G21.1, Tannenstrasse 3, 8092 Zürich, Switzerland, phone: +41 44 632 2447.

# **Supplementary material to the Materials and Methods section of the main manuscript**

## **Hardware**

The methods to control the pressure of the reservoirs via pressurized air and vacuum as well as to control the fluid level of the reservoirs by making use of the reflux pumps in the hydraulic part have been described earlier in detail.^9^ The hybrid mock circulation (HMC) operates as follows: The volume flow rates are measured and fed into the numerical model, which computes the pressures to be applied to the reservoirs in real time. These pressures of the hydraulic interfaces are applied to the active or passive cardiovascular implant tested and produce new volume flow rates, which are fed back to the numerical model, causing the loop to continue.

### *Active cardiovascular implant*

The control algorithm of the HVAD (Medtronic Inc., Minneapolis, MN, USA) motor was realized by using sensorless field-oriented control. The control unit was communicating with the data acquisition card of the HMC, thus setting the desired pump speed and recording the values of the actual speed and current. The speed can be set in the range of 1800 – 4000 rpm, similarly to the original control system.

## **Software**

### *Numerical model of the cardiovascular systems*

The numerical model of the cardiovascular system used in the current study constitutes a lumped-parameter model and relies on an earlier developed and validated model by Colacino et al.^3^ Various studies have used this model to investigate the interaction of a ventricular assist device (VAD) with a pathological circulation and to evaluate novel VAD algorithms.^1,7,8,10–12^ That model is depicted as an electrical equivalent circuit and consists of four main parts depicted in colors in Figure 2 of the main manuscript: the right heart (blue), the pulmonary circulation (light gray), the left heart (red) and the systemic circulation (dark gray).

In the case of **biventricular assist device (BiVAD)** support (P1 line in Figure 2), the models of the left and right heart consist of models for the atria, the ventricles and the valves. The atria are active and modeled by non-linear time-varying elastances. The ventricles are represented by non-linear time-varying elastances and internal resistances, as proposed by previous studies.^2,13,15^ The valves are modelled with a resistance and an inductor, as earlier proposed and validated.^13,15^ In the case of a positive pressure gradient across the valve, the resistance takes a low value and thus, a high flow and low pressure drop across the valve occur, similar to when the leaflets are open. In contrast, when the pressure gradient becomes negative, a high value is assigned to the valve resistance, thus mimicking the resistance of the valve with closed leaflets. The role of the inductor is to capture the dynamic behavior of the fluid inertia during the opening and closing of the leaflets. The VADs are connected in parallel to the ventricles.

In the case of **total artificial heart (TAH)** support (P2 line in Figure 2), the atria are passive and thus modeled by a constant elastance. The ventricles and valves are replaced with two separate VADs. In the case of **Fontan circulation** (P3 line in Figure 2), the left heart is similar to the BiVAD case, except from the fact that the systemic atrium has a constant elastance and the systemic ventricular resistance is also constant, as proposed by Granegger et al.^4^ The main difference exists on the elements of the right heart, which in this case are substituted by the physical model of the Fontan graft used.

In the **BiVAD** and **TAH** support cases, the pulmonary and systemic circulations are modelled based on the same approach. They are divided into the arterial- and venous-system lumped-parameter models, which include subgroups of arteries and veins, respectively. For the arterial systems, a five-element Noordergraaf model^14^ is used, which consist of a constant and a regulated resistance, an inductor and two constant elastances. The regulated systemic and pulmonary resistances are computed by the arterial baroreflex mechanisms. The latter are mimicked by transfer functions of first-order control systems.^9^ The venous system is modelled by a resistance and a compliance, based on Guyton’s model.^5^ For the pulmonary system, the resistance and the compliance are constant, while for the systemic system both are varying. A first-order controller controls the unstressed venous volume (V_0sv_ of Figure 2) of the venous systemic compliance, thus controlling the mean circulatory filling pressure of the circulation, which in turn regulates the cardiac output^5^ (“CO autoreg.” block of Figure 2). The varying resistance is also controlled by a first-order control system which mimics the venous resistance adaptation, as proposed by Guyton et al.^6^

In the **Fontan circulation** case, Granegger et al.^4^ modelled the arterial and venous systems with some modifications compared to Colacino et al.^3^ In particular, different arterial Windkessel models were used. Furthermore, the baroreflex mechanisms regulated both the arterial resistances and the shift of the unstressed volume to the stressed one, while no control mechanism for the systemic venous resistance was incorporated. In addition to those mechanisms, baroreflex mechanisms that regulate the maximum elastance of the systemic ventricle and the heart rate were implemented, based on the measured aortic pressure and its set value. Exercise was triggered by a decrease of systemic arterial resistance and increase of the set value for the arterial pressure.

The numerical models were implemented in MATLAB/Simulink running with Real-Time Windows Target (The Mathworks Inc., Natick, MA, USA). Two data acquisition boards (MF634 multifunction I/O card, Humusoft s.r.o., Prague, Czech Republic) were used for the in- and outputs of analog signals.

# **References**

1. Amacher, R., J. Asprion, G. Ochsner, H. Tevaearai, M. J. Wilhelm, A. Plass, A. Amstutz, S. Vandenberghe, and M. Schmid Daners. Numerical Optimal Control of Turbo Dynamic Ventricular Assist Devices. *Bioengineering* 22–46, 2014.doi:10.3390/bioengineering1010022

2. Campbell, K. B., J. A. Ringo, and N. S. Peterson. Sensitivity analysis of interaction between the left ventricle and the systemic arteries. In: Ventricular/Vascular Coupling. Springer, 1987, pp. 288–300.

3. Colacino, F. M., F. Moscato, F. Piedimonte, M. Arabia, and G. a. Danieli. Left Ventricle Load Impedance Control by Apical VAD Can Help Heart Recovery and Patient Perfusion: A Numerical Study. *ASAIO J.* 53:263–277, 2007.

4. Granegger, M., M. Schweiger, M. Schmid Daners, M. Meboldt, and M. Hübler. Cavopulmonary mechanical circulatory support in Fontan patients and the need for physiologic control: A computational study with a closed-loop exercise model. *Int. J. Artif. Organs* , 2018.doi:10.1177/0391398818762359

5. Guyton, A. C. Cardiac output and its regulation. *Circ. Physiol.* 353–371, 1973.

6. Guyton, A. C., T. G. Coleman, and H. J. Granger. Circulation: overall regulation. *Annu. Rev. Physiol.* 34:13–44, 1972.

7. Korn, L., D. Rüschen, N. Zander, S. Leonhardt, and M. Walter. Real-Time ECG Simulation for Hybrid Mock Circulatory Loops. *Artif. Organs* 0:, 2017.

8. Moscato, F., M. Arabia, F. M. Colacino, P. Naiyanetr, G. a Danieli, and H. Schima. Left ventricle afterload impedance control by an axial flow ventricular assist device: a potential tool for ventricular recovery. *Artif. Organs* 34:736–44, 2010.

9. Ochsner, G., R. Amacher, A. Amstutz, A. Plass, M. Schmid Daners, H. Tevaearai, S. Vandenberghe, M. J. Wilhelm, and L. Guzzella. A novel interface for hybrid mock circulations to evaluate ventricular assist devices. *IEEE Trans. Biomed. Eng.* 60:507–516, 2013.

10. Petrou, A., M. Monn, M. Meboldt, and M. Schmid Daners. A Novel Multi-objective Physiological Control System for Rotary Left Ventricular Assist Devices. *Ann Biomed Eng* 1–12, 2017.

11. Petrou, A., G. Ochsner, R. Amacher, P. Pergantis, M. Rebholz, M. Meboldt, and M. Schmid Daners. A Physiological Controller for Turbodynamic Ventricular Assist Devices Based on Left Ventricular Systolic Pressure. *Artif Organs* 40:842–855, 2016.

12. Rüschen, D., M. Rimke, J. Gesenhues, S. Leonhardt, and M. Walter. Online cardiac output estimation during transvalvular left ventricular assistance. *IFAC Proc. Vol.* 48:569–574, 2015.

13. Sun, Y., B. J. Sjoberg, P. Ask, D. Loyd, and B. Wranne. Mathematical model that characterizes transmitral and pulmonary venous flow velocity patterns. *Am. J. Physiol. Circ. Physiol.* 268:H476--H489, 1995.

14. Toy, S. M., J. Melbin, and A. Noordergraaf. Reduced models of arterial systems. *IEEE Trans. Biomed. Eng.* 174–176, 1985.

15. Vollkron, M., H. Schima, L. Huber, and G. Wieselthaler. Interaction of the Cardiovascular System with an Implanted Rotary Assist Device: Simulation Study with a Refined Computer Model. *Artif. Organs* 26(4):349–359, 2002.
